# Bardoxolone methyl ameliorates osteoarthritis by inhibiting osteoclastogenesis and protecting the extracellular matrix against degradation

**DOI:** 10.1016/j.heliyon.2023.e13080

**Published:** 2023-01-20

**Authors:** Ruijia Yang, Yanjing Guo, Sujing Zong, Zhou Ma, Zhenyu Wang, Jiyu Zhao, Jinmei Yang, Liping Li, Chongwei Chen, Shaowei Wang

**Affiliations:** a Shanxi Key Lab of Bone and Soft Tissue Injury Repair, Department of Orthopaedics, The Second Hospital of Shanxi Medical University, Taiyuan, China; b Department of Laboratory Medicine, Southern Central Hospital of Yunnan Province (The First People's Hospital of Honghe State), Mengzi, China; c Department of Biochemistry, Basic Medical College, Shanxi Medical University, Taiyuan, China; d Department of Pediatrics, Southern Central Hospital of Yunnan Province (The First People's Hospital of Honghe State), Mengzi, China

**Keywords:** Bardoxolone methyl, Osteoarthritis, Osteoclast, NF-κB, Nrf2/HO-1

## Abstract

Inflammation and oxidative damage are closely related to the development of osteoarthritis. Bardoxolone methyl (CDDO-Me), a semisynthetic oleanane triterpenoid, plays a strong anti-inflammatory and antioxidant role. The purpose of our research was to explore fundamental mechanisms of CDDO-Me in orthopaedics development. The results showed that CDDO-Me inhibited nuclear factor-κB ligand (RANKL)-induced osteoclast formation and extracellular matrix (ECM) degradation by activating the Nrf2/HO-1 signaling pathways and inhibiting NF-κB pathway activation and excess ROS production. In vivo, CDDO-Me significantly attenuated articular cartilage proteoglycan loss and the number of TRAP-positive osteoclasts in a destabilized medial meniscus (DMM) mouse model of OA. Taken together, these data demonstrate that CDDO-Me inhibits osteoclastogenesis and ECM degradation, underscoring its potential therapeutic value in treating OA.

## Graphical abstract

**Figure:**
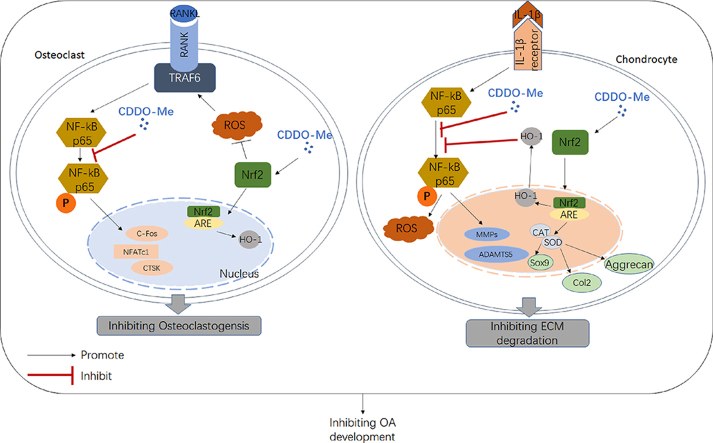

## 1 Introduction

Osteoarthritis (OA) is a prevalent degenerative joint disorder characterized by cartilage degeneration, and the incidence of this disease increases with age [1]. Sex, trauma, mechanical stress, and genetics are also risk factors for osteoarthritis, but there are few effective treatments for OA [[2], [3], [4]]. The main pathological features of OA include loss of cartilage, imbalance in extracellular matrix synthesis and metabolism, inflammation, oxidative stress, and subchondral bone sclerosis [[5], [6], [7]]. The degradation of cartilage extracellular matrix by metalloproteinases leads to the development of OA [5]. Interleukin-1 beta (IL-1β), an inflammatory cytokine, has a significant proinflammatory effect on the pathogenesis of OA and activates multiple signaling pathways [8]. IL-1β inhibits type II collagen expression and increases the production of matrix metalloproteinases (MMPs) and a disintegrin-like metalloproteinase with thrombospondin motif (ADAMTS), which promote extracellular matrix (ECM) degradation, ultimately contributing to OA progression [9].

Articular cartilage degeneration is the main feature of OA, but subchondral bone is attracting increasing attention and plays an important role in the pathogenesis of early-stage OA [10]. During OA progression, subchondral bone loss drives the increase in bone remodeling and overactivation of osteoclasts, which induce catabolism of articular cartilage and promote the development of osteoarthritis [11,12]. Osteoprotegerin (OPG), an inactivating receptor for RANKL, binds to RANKL and inhibits osteoclasts. When mice are treated with OPG, cartilage deterioration is largely prevented [13]. The degradation of articular cartilage and remodeling of subchondral bone during OA reflect the abnormal state of chondrocytes and osteoclasts [14,15]. This finding suggests that targeted inhibition of osteoclast formation and maintaining the balance of ECM metabolism in chondrocytes may be an effective strategy to treat osteoarthritis.

Nuclear factor-kappa B (NF-κB), an inflammatory mediator, is abnormally activated in OA. NF-κB induces catabolic gene expression and promotes the expression of major proinflammatory and destructive mediators of OA, leading to chondrocyte catabolism and synovial inflammation [9,16]. Some research has shown that the NF-κB pathway, which can be activated by IL-1β to induce ECM catabolism, leads to OA onset and progression [17]. In addition, macrophage colony-stimulating factor (M-CSF) and nuclear factor receptor-activating factor (NF)-κB ligand (RANKL)-induced bone marrow macrophages (BMMs) can differentiate into osteoclasts (OCs) [18]. The NF-κB pathway can be activated by RANKL during osteoclast differentiation [19]. NF-κB plays an important role in both chondrocytes and osteoclasts. Increasing evidence has shown that NF-κB can be inhibited by Nrf2 to alleviate OA [20,21].

Nuclear factor erythroid derived-2-related factor 2 (Nrf2) is the master regulator of cellular redox homeostasis. Nrf2 translocates to the nucleus and then activates the expression of antioxidant genes such as heme oxygenase-1 (HO-1) to decrease ROS levels and suppress the phosphorylation of NF-ĸB p65, ultimately ameliorating OA [20]. It was found that Nrf2 knockout exacerbates OA, which is further evidence of the important role of Nrf2 in the pathogenesis of OA [22]. Furthermore, Nrf2 is closely associated with osteoclast formation [23].

Excessive production of reactive oxygen species (ROS) leads to progressive mitochondrial damage in chondrocytes and disrupts normal matrix repair [24]. ROS are closely associated with osteoclast formation. Studies have confirmed that Nrf2 can decrease ROS levels to inhibit OC formation [25]. Thus, activation of the Nrf2/HO-1 pathway to decrease chondrocyte oxidative damage, inhibit imbalanced metabolism mediated by the NF-κB pathway and block osteoclast formation is a promising research direction for OA treatment.

At present, the main treatments for osteoarthritis are pharmacological and nonpharmacological interventions (such as exercise and weight loss) [14]. Nonsteroidal anti-inflammatory drugs (NSAIDs), corticosteroids, and hyaluronic acid target pain reduction or functional improvements in OA and are commonly used in clinical practice to treat OA [26]. However, there are notable safety issues associated with the long-term use of these drugs, which only provide short-term relief without preventing or reversing cartilage degeneration [27].

**Fig. 1:**
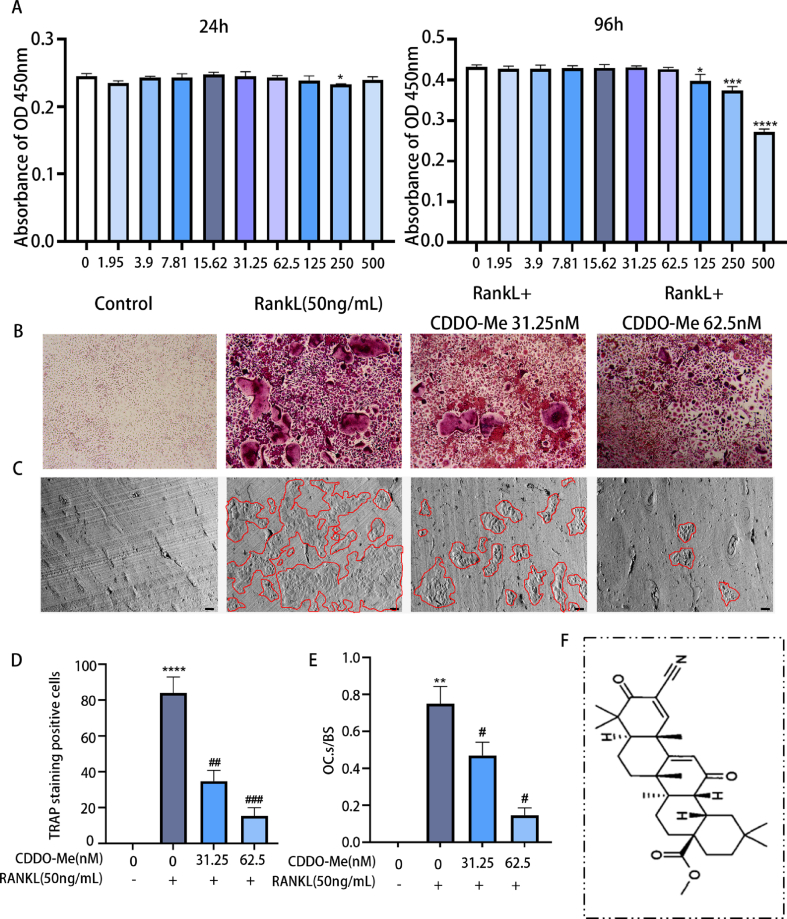
CDDO-Me dose-dependently suppresses osteoclastogenesis induced by RANKL. n = 3. (A) Assesses of the cytotoxicity of CDDO-Me on BMMs by the CCK-8 assay at 24 h and 96 h. (B) TRAP staining of OCs treated with CDDO-Me. (C) Images of the bone slice resorption areas, scale bar, 100 μm. (D) Quantification of TRAP positive osteoclast cells including 3 or more nuclei. n = 3. (E) The bone slice resorption areas were quantitatively analyzed n = 3. (F) The molecular structure of CDDO-Me. *P < 0.05, **P < 0.01, ***P < 0.001 and ****P < 0.0001. ^##^P < 0.01 and ^###^P < 0.001 compared with RANKL group.

Triterpenoids are abundant in nature and have high medicinal value [28]. Bardoxolone methyl (CDDO-Me) is a newly discovered semisynthetic oleanane triterpenoid derivative based on naturally occurring triterpenoids; and its activity is stronger than that of natural triterpenes [28] (for the chemical structure of CDDO-Me, see Fig. 1F). CDDO-Me was confirmed to exert anti-inflammatory, antioxidant, anti-cancer, anti-fibrotic and anti-viral effects [[29], [30], [31], [32]]. Besides, CDDO-Me plays an anti-inflammatory and antioxidant effects by inhibiting the NF-KB pathway and activating the Nrf2/HO-1 pathway [33]. It has been reported that CDDO-Me is often used clinically to treat nephritis, liver disease, and pancreatic cancer [32,34,35]. However, there have been few reports about the effects of CDDO-Me on OA.

In the present study, we found that CDDO-Me attenuated OA progression by inhibiting cartilage extracellular matrix metabolism and osteoclastogenesis by activating the Nrf2/HO-1 pathway to reduce ROS production and inhibit the NF-κB pathway.

## 2 Materials and methods

### 2.1 Primary cell culture

Primary bone marrow macrophages (BMMs) were isolated from the femur and tibia of 6-week-old C57BL/6 mice and cultured in α-modified minimal essential medium (α-MEM, Gibco, USA) containing 10% fetal bovine serum (Gibco, USA) and 1% penicillin and streptomycin (Thermo Fisher), and 25 ng/mL of macrophage-colony stimulating factor (M-CSF). BMMs were incubated at 37 °C with 5% CO_2_, and the medium was changed every 2 days. The study was reviewed and approved by the Ethical Committee of Shanxi Medical University.

### 2.2 Osteoclast differentiation

BMMs were seeded in 96-well plates at a concentration of 8 × 10^3^ cells per well in α-MEM containing 25 ng/mL M-CSF (LifeTein, LLC., Beijing, China) overnight. The BMMs were stimulated with 100 ng/mL RANKL (LifeTein, LLC., Beijing, China) after adherence. Moreover, various concentrations of CDDO-Me (0 nM, 3.9 nM, 7.8 nM, 15.6 nM, 31.2 nM, 62.5 nM) were added to the cultured cells. The media, which contained 25 ng/mL M-CSF and 100 ng/mL RANKL, were replaced every 2 days, Furthermore, media containing CDDO-Me was changed every 2 days until the Day 7, when OCs formed. Tartrate-resistant acid phosphatase (TRAP) levels in differentiated OCs were evaluated after the cells were fixed with 4% paraformaldehyde for 1 h. OC differentiation was evaluated by counting the number of TRAP-positive multinucleated cells with 3 or more nuclei.

### 2.3 Cell viability assessment

The cells were cultured in DMEM/F12 medium (Gibco, USA) containing 10% fetal bovine serum (Gibco, USA) and 1% penicillin and streptomycin (Thermo Fisher Scientific, USA). Cell viability was measured using a CCK-8 kit (JoyTech Bio Ltd., China) according to the instructions. In brief, ATDC5 cells (5 × 10^3^ cells/well) were cultured in 96-well plates at 37 °C in a 5% CO_2_ incubator overnight. Cells in the IL-1β (Cranbury, NJ, USA) group and control group were cultured in different concentrations of CDDO-Me (0 μM, 0.1 μM, 0.2 μM, 0.4 μM, 0.6 μM, 0.8 μM, and 1.2 μM) for 24, 48, 72 and 96 h. Then, the cells were washed with PBS, and 100 μL of serum-free DMEM/F12 containing 10 μL of CCK-8 solution was added to each well and incubated for 2 h at 37 °C. Finally, an enzyme labeling instrument (Thermo Fisher Scientific, USA) was used to measure the absorbance at 450 nm.

BMMs were seeded in plates (96-well plate) at density of 3 × 10^3^ cells/well for 24 h and treated with differed concentrations of CDDO-Me (0 nM, 7.8 nM, 15.6 nM, 31.2 nM, 62.5 nM, 125 nM) for 24, 48, 72, 96 h, and the next steps were the same those described for ATDC5 cells.

### 2.4 ROS assessment

ROS levels were assessed with a DCFH-DA probe in ATDC5 cells and BMMs according to the manufacturer's instructions (Solarbio, Beijing, China). ATDC5 cells were seeded in 6-well plates at a density of 8 × 10^4^ cells/well. The cells were treated with 0.6 μM CDDO-Me for 2 h and then incubated with 10 μM DCFH-DA in the dark for 20 min at 37 °C. The cells were washed three times with PBS, and finally, 10 ng/mL IL-1β was added and incubated for 10 min, after which the cells were washed three times with PBS.

BMMs were seeded at a density of 8 × 10^6^ cells per well. BMMs were treated with 25 ng/mL M-CSF, 100 ng/mL RANKL and 62.5 nM CDDO-Me for 3 days and then were incubated with 10 μM DCFH-DA in the dark for 20 min at 37 °C, after which the BMMs were washed with PBS (Solarbio, Beijing, China). The number of ROS-positive cells was examined by fluorescence microscopy. The fluorescence intensity was quantified using ImageJ.

### 2.5 High density culture

ATDC5 cells were seeded at a density of 2 × 10^4^ cells in a volume of 10 μL of DMEM/F12 containing 10% (w/v) FBS in the middle of a 24-well plate. The cells were allowed to adhere for 1 h at 37 °C, and this process was repeated four times. Eventually, 500 μL of medium was added to each well and incubated overnight at 37 °C. After that, different concentrations of CDDO-Me (0 μM, 0.2 μM, 0.4 μM, 0.6 μM) were added to wells in the IL-1β group and control group. The medium was refreshed every other day, and after 7 days, the micromasses were fixed in 4% paraformaldehyde for 40 min and processed for Alcian Blue staining (Solarbio, Beijing, China) for 1 h.

### 2.6 Human cartilage explant culture

“Relatively healthy” cartilage samples were collected from the tibial plateau during knee arthroplasty. Human articular tissue was dissected from the joint surface and cut into 4 mm^3^ explants. All cartilage explants were randomly divided into the control and IL-1β groups and cultured in DMEM/F12 supplemented with 10% FBS and CDDO-ME (same concentrations of CDDO-Me as in the high-density culture). After 24 h, 48 h, and 72 h of treatment, the samples were fixed with 4% paraformaldehyde for 24 h, histological sections were prepared after the samples were dehydrated and embedded. The severity of OA was assessed by histological observation using safranin-O/fast green staining (Solarbio, Beijing, China). In addition, immunohistochemistry was used to measure the protein expression levels of MMP13 (1:100, Cat. #AF5355, Affinity) and Col2a (1:100, ab34712, Abcam).

### 2.7 Real-time quantitative polymerase chain reaction (RT–qPCR)

ATDC5 cells (8 × 10^4^ cells per well) were seeded in 6-well plates and incubated for 24 h at 37 °C. CDDO-Me was added to the control and IL-1β groups (the CDDO-Me concentration was the same as described above), and the culture medium was replaced every day for 7 days. We extracted total RNA from the cells using TRIzol reagent (TaKaRa, Dalian, China), and then PrimeScript RT Master Mix (TaKaRa, Dalian, China) was used to reverse-transcribe cDNA according to the manufacturer's protocol. qPCR was performed using a TB Green Premix Ex Taq kit (TaKaRa, Dalian, China) on an Applied Biosystems QuantStudio 6 Flex Real-Time PCR system (Thermo Fisher Scientific, Waltham, MA, USA). The primer sequences for 18S, Sox9, Aggrecan, Nrf2, SOD1, SOD2, Cat, ADAMTS5, MMP3 and MMP13 are listed in Table 1.

**Table 1:** Primer sequences used in RT-qPCR.

Gene	Froward (5′-3′)	Reverse (5′-3′)
Sox9	GAGCCGGATCTGAAGAGGGA	GCTTGACGTGTGGCTTGTTC
Aggrecan	CAGTGGGATGCAGGCTGGCT	CCTCCGGCACTCGTTGGCTG
ADAMTS5	GGAGCGAGGCCATTTACAAC	CGTAGACAAGGTAGCCCACTTT
MMP3	ACATGGAGACTTTGTCCCTTTTG	TTGGCTGAGTGGTAGAGTCCC
MMP13	CTTCTTCTTGTTGAGCTGGACTC	CTGTGGAGGTCACTGTAGACT
Nrf2	TTCCTCTGCTGCCATTAGTCAGTC	GCTCTTCCATTTCCGAGTCACTG
SOD1	ATTCACTTCGAGCAGAAGGCA	ATTGCCCAGGTCTCCAACAT
SOD2	GTGTCTGTGGGAGTCCAAG	TGCTCCCACACATCAATCCC
CAT	AGAGGAAACGCCTGTGTGAG	TAGTCAGGGTGGACGTCAGT
CTSK	CCAGTGGGAGCTATGGAAGA	AAGTGGTTCATGGCCAGTTC
DC-STAMP	TTCATCCAGCATTTGGGAGT	ACAGAAGAGAGCAGGGCAAC
AcP5	CAGCAGCCAAGGAGGACTAC	ACATAGCCCACACCGTTCTC
NFATC1	CAACGCCCTGACCACCGATAG	GGCTGCCTTCCGTCTCATAGT
c-Fos	TTTCAACGCCGACTACGAGG	GCGCAAAAGTCCTGTGTGTT
TNFRSF11	GAAGATGCTTTGGTGGGTGT	TCAGTCGGGATCAGTGTGAG
18S	CGGCTACCACATCCAAGGAA	GCTGGAATTACCGCGGCT

BMMs (13 × 10^5^ cells/well) were grown in 6-well plates and cultured in medium containing 10% (v/v) FBS, M-CSF (25 ng/mL), and RankL (100 ng/mL) with or without different concentrations of CDDO-Me (62.5 nM, 31.2 nM) for 5 days. Total RNA was extracted from the cells as described previously. The target genes 18S, Nrf2, SOD1, SOD2, CTSK, DC-STAMP, AcP5, NFATC1, c-Fos, and TNFRSF11 were examined. The sequences are listed in Table 1.

Finally, the relative target gene expression level was evaluated by the 2^−ΔΔCq^ method [36]. The specific methods are as follows: Firstly, the CT of each group of target genes minus the CT of the internal reference genes was calculated as ΔCT, and secondly, the normal group without IL-1 and CDDO-Me or without RANKL and CDDO-Me treatment was taken as the control group, and the ΔCT of the gene of each experimental group minus the ΔCT of the corresponding gene of the control group was calculated as ΔΔCT, and finally, 2^−ΔΔCT^ was calculated.

### 2.8 Western blot analysis

To assess the expression of proteins related to cartilage synthesis and metabolism, ATDC5 cells were seeded in 6-well plates (12 × 10^4^ cells/well) overnight for adherence. Then, CDDO-Me (0.2 μM, 0.4 μM, 0.6 μM) was added to the control and IL-1β groups, and total proteins were harvested at 48 h. A total of 20 × 10^4^ cells per well was incubated overnight in serum-free medium after being pretreated with CDDO-Me (0.2 μM, 0.4 μM, 0.6 μM) for 2 h at 37 °C. Then the cells were stimulated with IL-1β for 30 min, and total cellular proteins were extracted in order to evaluate the Nrf2/Ho-1 and NF-κB pathways. RIPA lysis buffer (Boster, Wuhan, China) containing phosphatase and protease inhibitors was used to extract the proteins.

SDS–PAGE (10%) was used to separate proteins by size, and then the protein samples were transferred to a polyvinylidene fluoride (PVDF) membrane and blocked for 2 h with 5% skimmed milk in Tris-buffered saline (TBS) with 0.1% Tween-20 (TBST). The membranes were incubated overnight at 4 °C with the following primary antibodies: anti-MMP13 (1:1000, Cat.# AF5355, Affinity), anti-MMP3 (1:1000, Cat.# AF0217, Affinity), anti-Aggrecan (1:1000, sc-166,951, Santa Cruz), anti-Sox9 (1:1000, ab185966, Abcam), anti-p65 (1:1000, Cat.# BF0382, Affinity), anti-phospho-p65 (1:1000, Cat.# AF2006, Affinity), anti-Nrf2 (1:1000, Cat.# AF0639, Affinity), anti–HO–1 (1:1000, ab52947, Abcam), anti-*c*-Fos (1:1000, Cat.# AF0132, Affinity), anti-NFATc1 (1:1000, sc-7294, Santa Cruz), anti-CTSK (1:1000, sc-48353, Santa Cruz), anti-Integrinβ3 (sc-365679, Santa Cruz), anti-GAPDH (1:1000, Cat. AC001, Abconal), and anti-βactin (1:1000, Cat. AC026, Abconal). Then, the membranes were incubated with anti-rabbit (1:2000, RS0001, Immunology) or anti-mouse (1:2000, RS0002, Immunology) secondary antibodies for 2 h at room temperature. Finally, the immunoreactive bands were visualized by the Touch Imaging System by Bio-Rad (ChemiDoc™, Bio–Rad, CA, USA) according to the manufacturer's instructions.

### 2.9 Destabilized medial meniscus (DMM) mouse model

Twenty-four 8-week-old C57BL/6 male mice were purchased from the Animal Center of Shanxi Medical University (Shanxi, China). For the DMM-induced osteoarthritis model, surgery was performed on the right knees of eighteen mice as previously described, severing the medial meniscus tibial ligament causes instability of the medial meniscus, leading to damage to the articular cartilage and eventually to osteoarthritis [37]. After a surgery, the mice were randomly divided into four groups: sham group, DMM group, DMM plus 10 mg kg^−1^ CDDO-Me group, and DMM plus 50 mg kg^−1^ CDDO-Me group. CDDO-Me was administered intraperitoneally to DMM mice for 8 consecutive weeks after surgery. After 8 weeks, the mice were sacrificed, and the subchondral bone of the tibia was analyzed by histology and micro-CT.

### 2.10 Micro-CT analysis

We measured the following parameters of mouse tibia samples: bone volume/tissue volume (BV/TV), bone surface/volume fraction (BS/BV), structure model index (SMI), trabecular number (Tb.N), trabecular separation (Tb.Sp) and trabecular thickness (Tb.Th). We dissected the right knee joints free of soft tissue, fixed the samples overnight in 4% paraformaldehyde, and scanned them with high-resolution μCT. The scanner was set at a voltage of 50 kV, a current of 200 μA, and a resolution of 5.7 μm per pixel. Then, we subjected the subchondral bone region to analysis.

### 2.11 Histochemistry and immunohistochemistry

All mouse right tibia samples were fixed in 4% paraformaldehyde for 48 h and then decalcified by 14% EDTA at 37 °C for 14 days. Five-micrometer-thick sagittal sections of the knee joint were processed for hematoxylin and eosin (H&E), safranin O-fast green and tartrate-resistant acid phosphatase (TRAP) staining. Immunohistochemistry was performed using a standard protocol. We incubated the sections overnight at 4 °C with the following primary antibodies: MMP13 (1:100, Cat.# AF5355, Affinity), Col2a (1:100, ab34712, Abcam), anti-phospho-p65 (1:1000, Cat.# AF2006, Affinity), and anti-Nrf2 (1:1000, Cat.# AF0639, Affinity). Then, the cells were incubated with anti-rabbit secondary antibodies for 1 h at 37 °C and visualized with DAB (ZSGB-BIO, Beijing, China). Finally, the OARSI score was used to assess cartilage damage and quantify TRAP-positive cells and immunopositive cells using ImageJ.

### 2.12 Immunofluorescence analysis

The proteins Nrf2 and PP65 were assessed in vitro. ATDC5 cells were seeded in 6-well plates and divided into the control, IL-1β, and IL-1β plus CDDO-Me (0.6 μM) groups. ATDC5 cells were pretreated with CDDO-Me for 24 h followed by stimulation with IL-1β (10 ng/mL) for 30 min. To assess Sox9 and MMP13 expression, ATDC5 cells were divided into the control, CDDO-Me (0.6 μM), IL-1β, and IL-1β plus CDDO-Me (0.6 μM) groups. The cells were treated with CDDO-Me (0.6 μM) or IL-1β or were treated with IL-1β plus CDDO-Me for 48 h. The cells were washed three times in PBS and then fixed with 4% paraformaldehyde for 30 min, followed by permeabilization using 0.1% Triton X-100 diluted in PBS for 20 min. Then, the sample was blocked with goat serum for 1 h at 37 °C and incubated overnight at 4 °C with the following primary antibodies: anti-MMP13 (1:1000, Cat.# AF5355, Affinity), anti-Sox9 (1:1000, ab185966, Abcam), anti-phospho-p65 (1:1000, Cat.# AF2006, Affinity), and anti-Nrf2 (1:1000, Cat.# AF0639, Affinity). Next, the samples were incubated with fluorescent secondary antibodies (1:100, Cat. BA1127, Boster) for 1 h at room temperature and labeled with DAPI (Cat. AR1176, Boster) for 30 min. Finally, the slide was sealed with 50% glycerin, and the fluorescence intensity was measured using ImageJ.

### 2.13 Bone resorption assay

Bovine bone slices were placed into 96-well plates, then BMMs were seeded in 96-well plates and cultured in α-MEM including 25 ng/mL M-CSF for 24 h; after adherence, the medium in 96-well was changed. The α-MEM medium containing 25 ng/mL M-CSF, 100 ng/mL RANKL, and different concentrations of CDDO-Me (0, 31.25, 62.5 nM). After changing the medium every other day for 14 days, the slides were washed with 75% alcohol and trypsin, after which the bone slices were cleaned with ddH_2_O and brush to completely remove cells. Then, the bone slices were completely dried in a 70 °C oven. Bone resorption pits were observed using scanning electron microscopy (TM-1000; Hitachi, Tokyo, Japan) and measured the gross resorption pit areas using ImageJ software.

### 2.14 Statistical analysis

The data are expressed as the means ± standard deviation (SD). One-way ANOVA was followed by Tukey's post-hoc test was performed GraphPad Prism 8. p < 0.05 was indicated to be significant.

## 3 Results

### 3.1 CDDO-Me inhibits RANKL-induced osteoclastogenesis

CCK-8 experiment data indicated that concentrations of CDDO-Me including 31.25 nM and 62.5 nM did not inhibit the proliferation of BMMs after 24,48,72 and 96 h (Fig. 1A and Supplementary S1) (24 h, 250 nM p = 0.0119; 96 h, 125 nM p = 0.0232; 250 nM p = 0.0006; 600 nM p < 0.0001). Next, we assessed whether CDDO-Me could inhibit the differentiation of OCs, and TRAP staining was used. As shown in Fig. 1B, D, there was a dose-dependent decrease in the number of TRAP-positive cells. There were markedly fewer TRAP-positive cells in wells treated with 31.25 nM and 62.5 nM CDDO-Me than in untreated wells (RANKL groups, p < 0.0001; 31.25 nM, p = 0.0014; 62.5 nM, p = 0.0003). Then BMMs were seeded on bovine bone slices and treated them with RANKL and different CDDO-Me concentrations (31.25 and 62.5 nM) to assess the bone resorption activity of OCs. Compared to the control, the non-treatment group, we observed significant increase in bone resorption pit numbers following RANKL induce, but the resorption pit numbers was decreased in a dose-dependent manner when treated with CDDO-Me (Fig. 1C, E) (RANKL groups, p = 0.0052; 31.25 nM p = 0.0327; 62.5 nM p = 0.0144). These data suggest that CDDO-Me inhibits OC differentiation in a dose-dependent manner.

### 3.2 CDDO-Me reduces the expression of osteoclast genes

**Fig. 2:**
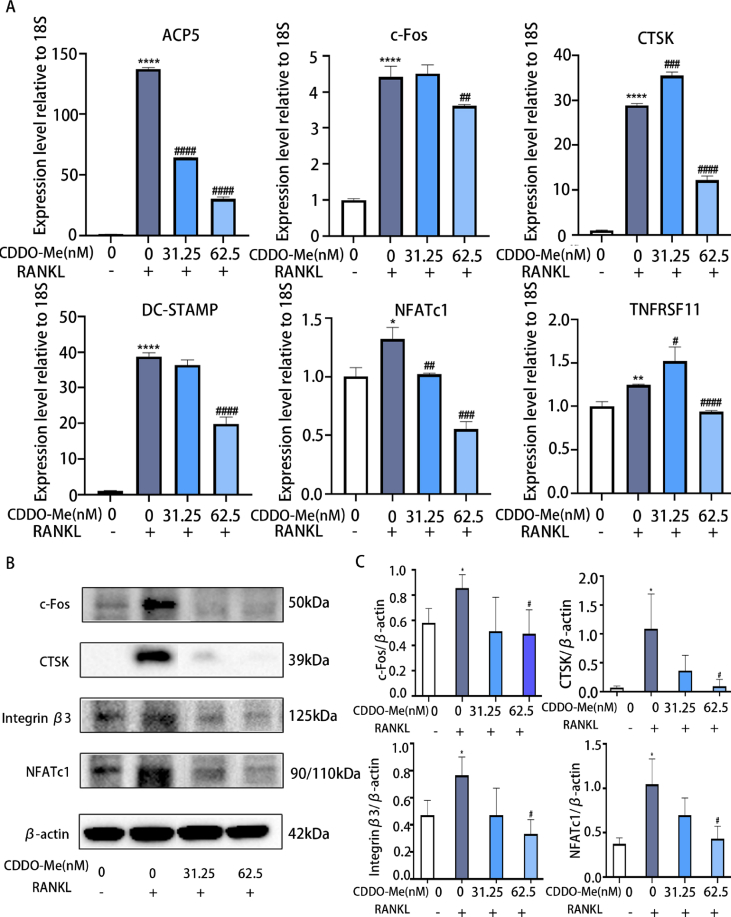
Osteoclastogenesis-related genes are down-regulated by CDDO-Me. (A) The expression of osteoclast specific mRNA, involving ACP5, c-Fos, CTSK, DC-STAMP, NFATc1, TNFRSF11 was analyzed by PT-qPCR. 18S was selected as the control gene, n = 3. (B) Western blots analysis of the integrin β3, CTSK, NFATc1, and c-Fos expression induced by treatment with RANKL (50 ng/mL) for 5 days in the presence or absence of CDDO-Me (31.25 nM and 62.5 nM). See supplementary material for non-adjusted images. (C) The quantification of band intensities corresponding to c-Fos, CTSK, NFATC1, integrin β3. Theβ-actin was selected as the control protein, n = 3. *P < 0.05, **P < 0.01 and ****P < 0.0001 compared with control group. ^#^P < 0.05, ^##^P < 0.01, ^###^P < 0.001 and ^####^P < 0.0001 compared with RANKL group.

To investigate the effect of CDDO-Me on RANKL-induced OC differentiation and function, we analyzed the expression of OC formation-related genes (NFATc1 and c-Fos), fusion-related genes (DC-STAMP), function-related genes (Acp5 and CTSK) and the TNFRSF11 gene, which encodes the RANKL protein, by qRT–PCR. The results showed that compared with those in the control group, the gene expression levels of NFATc1, c-Fos, DC-STAMP, Acp5, CTSK and TNFRSF11 in the RANKL-treated group were substantially elevated. In contrast, the expression of these genes was downregulated by CDDO-Me treatment. However, the effect of 31.25 nM CDDO-Me on c-FOS, CTSK, and TNFRSF11 was not significant (Fig. 2A). (ACP5: all group, p < 0.0001; c-Fos: RANKL groups, p < 0.0001; 62.5 nM, p = 0.0094; CTSK, RANKL groups and 52.5 nM, p < 0.001; 31.25 nM, p = 0.0002; DC-STAMP: RANKL groups and 62.5 nM, p < 0.0001; NFATc1: RANKL groups, p = 0.0113; 31.25 nM, p = 0.0063; 62.5 nM, p = 0.0003; TNFRSF11: RANKL groups p = 0.0014; 31.25 nM, p = 0.0451; 62.5 nM, p < 0.0001). These results confirmed that CDDO-Me suppressed RANKL-induced differentiation and function in OCs. Next, we verified the expression of osteogenesis- and function-related proteins, including NFATc1, c-Fos and downstream proteins, by Western blotting. As shown in Fig. 2B, the expression of c-Fos, NFATc1, integrin β3 and CTSK was significantly increased in the RANKL-induced group. Similarly, 31.25 nM and 62.5 nM CDDO-Me inhibited RANKL-induced OC formation and function, and the effect was dose-dependent, which was consistent with the fold change and qRT–PCR results (Fig. 2C) (c-Fos: RANKL groups, p = 0.0367; 62.5 nM, p = 0.0450; CTSK: RANKL groups, p = 0.0431; 62.5 nM, p = 0.0488; integrin β3: RANKL groups, p = 0.0428; 62.5 nM, p = 0.0119; NFATc1: RANKL groups, p = 0.0165; 62.5 nM, p = 0.0288). The qRT–PCR and Western blot results showed that CDDO-Me effectively inhibited critical genes and proteins associated with OC differentiation and downstream OC function.

### 3.3 CDDO-Me promotes cartilage matrix synthesis and inhibits IL-1β-induced metabolism of cartilage matrix in ATDC5 cells

**Fig. 3:**
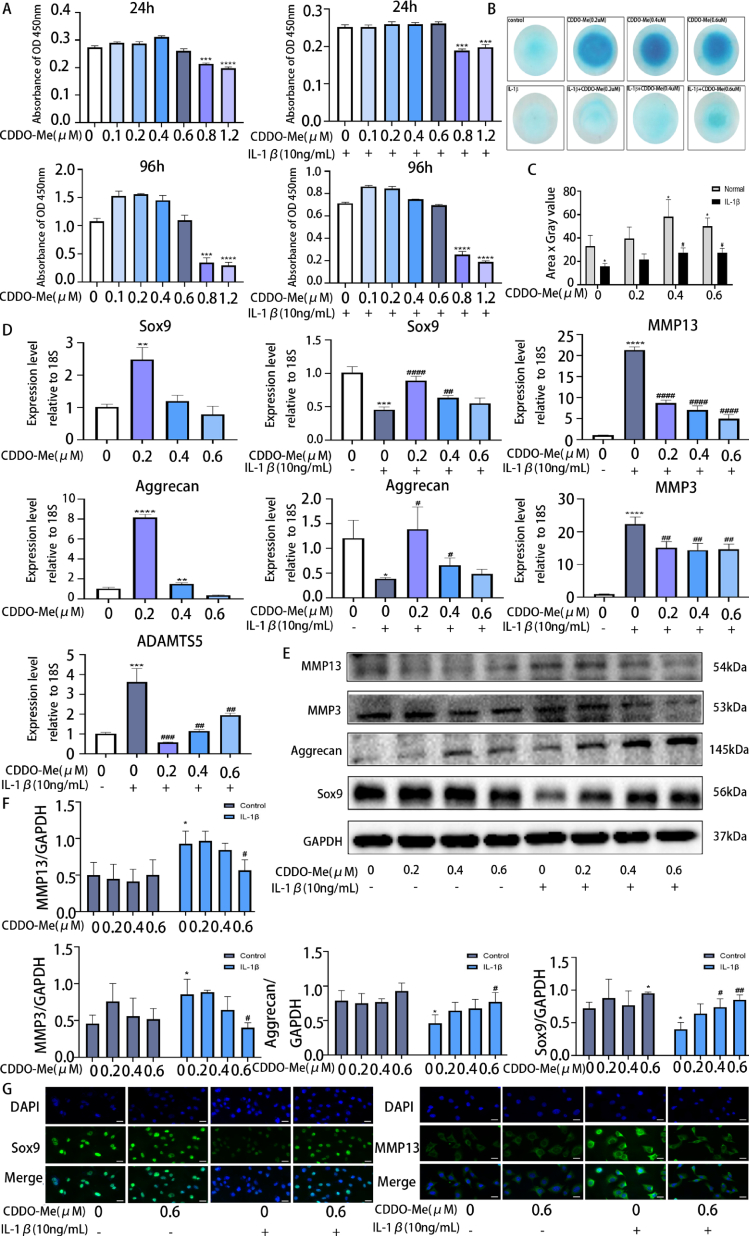
CDDO-Me promotes cartilage matrix synthesis and inhibits IL-1β-induced extracellular matrix degradation in ATDC5 cells. (A) CCK-8 assay of CDDO-Me cytotoxicity on ATDC5 cells at 24 h and 96 h, n = 3. (B, C) ATDC5 cells cultured at high density for 7 days for Alcian blue staining and circle intensities was quantified, n = 3. (D) RT-qPCR was used to assess the expression of chondrocyte synthesis and degradation genes, including Sox9, Aggrecan, MMP13, MMP3, ADAMTS5. 18S was selected as the control gene, n = 3. (E) Western blots analysis of the Sox9, Aggrecan, MMP13, MMP3 expression induced by treatment with or without IL-1β (10 ng/mL) for 2 days in the presence or absence of CDDO-Me (0.2 μM, 0.4 μM, and 0.6 μM). See supplementary material for non-adjusted images. (F) The quantification of Sox9, Aggrecan, MMP13, MMP3. The GAPDH was selected as the control protein, n = 3. (G) The representative Sox9 and MMP13 were detected by the immunofluorescence combined with DAPI staining for nuclei (scale bar: 20 μm). *P < 0.05, **P < 0.01, ***P < 0.001 and ****P < 0.0001 compared with control group. ^#^P < 0.05, ^##^P < 0.01, ^###^P < 0.001 and ^####^P < 0.0001 compared with IL-1βgroup. (For interpretation of the references to color in this figure legend, the reader is referred to the Web version of this article.)

CCK-8 assay showed that 0.2 μM, 0.4 μM and 0.6 μM CDDO-Me had no significant toxic effects on ATDC5 cells, after 24,48,72 and 96 h (Fig. 3A and Supplementary S1) (24 h: 0.8 μM, p = 0.0001; 1.2 μM, p < 0.0001; 24 h IL-1β: 0.8 μM, p = 0.0001; 1.2 μM, p = 0.0006; 96 h: 0.8 μM, p = 0.0002; 1.2 μM, p < 0.0001; 96 h IL-1β: 0.8 μM, p < 0.0001; 1.2 μM, p < 0.0001). Next, to examine the effect of CDDO-Me on the synthesis and catabolism of extracellular matrix in ATDC5 cells. Alcian blue staining indicated that dose-dependently deepened in response to 0.2 μM, 0.4 μM and 0.6 μM CDDO-Me compared with the control group (Fig. 3B and C) (Normal:0.4 μM, p = 0.0433; 0.6 μM, p = 0.0415; IL-1β: 0 μM, p = 0.0353; 0.4 μM, p = 0.0139; 0.6 μM, p = 0.0100). To further investigate the mechanism of CDDO-Me, the expression of indicators ECM anabolic were increased in normal or IL-1β-stimulated conditions, and indicators ECM catabolic were suppressed in IL-1β-induced conditions, including Sox9, Aggrecan, MMP13, MMP3, ADAMTS5 were analyzed by qRT-PCR. Compared with that in the control, the expression of indicators ECM anabolic were increased in normal or IL-1β-stimulated conditions, and indicators ECM catabolic were suppressed in IL-1β-induced conditions (Fig. 3D) (Sox9: 0.2 μM, p = 0.0027; Sox9 IL-1β: 0 μM, p = 0.0001; 0.2 μM, p < 0.0415; 0.4 μM, p = 0.0022; Aggrecan: 0.2 μM, p < 0.0415; 0.4 μM, p = 0.0017; Aggrecan IL-1β: 0 μM, p = 0.0169; 0.2 μM, p = 0.0183; 0.4 μM, p = 0.0327; MMP13: all group, p < 0.0001; MMP3: 0 μM, p < 0.0001; 0.2 μM, p = 0.0026; 0.4 μM, p = 0.0019; 0.6 μM, p = 0.0039; ADAMTS5: 0 μM, p = 0.0003; 0.2 μM, p = 0.0007; 0.4 μM, p = 0.0017; 0.4 μM, p = 0.0093). Moreover, we analyzed anabolic and catabolic indices at the protein level. The Western blot results showed that the expression of the synthesis-related proteins Sox9 and Aggrecan increased, and the expression of the catabolic proteins MMP13 and MMP3 decreased (Fig. 3E and F) (MMP13 IL-1β: 0 μM, p = 0.0380; 0.6 μM, p = 0.0039; MMP3 IL-1β: 0 μM, p = 0.0419; 0.6 μM, p = 0.0212; Aggrecan IL-1β: 0 μM, p = 0.0409; 0.6 μM, p = 0.0428; Sox9: 0.6 μM, p = 0.0138; Sox9 IL-1β: 0 μM, p = 0.0164; 0.6 μM, p = 0.0036). The immunofluorescence staining results were consistent with the Western blot results (Fig. 3G). These results confirm that CDDO-Me promotes cartilage matrix synthesis and inhibits IL-1β-induced metabolism of the cartilage matrix.

### 3.4 CDDO-Me suppresses the NF-κB signaling pathway in both RANKL-induced osteoclastogenesis and IL-1β-induced ATDC5 cells

**Fig. 4:**
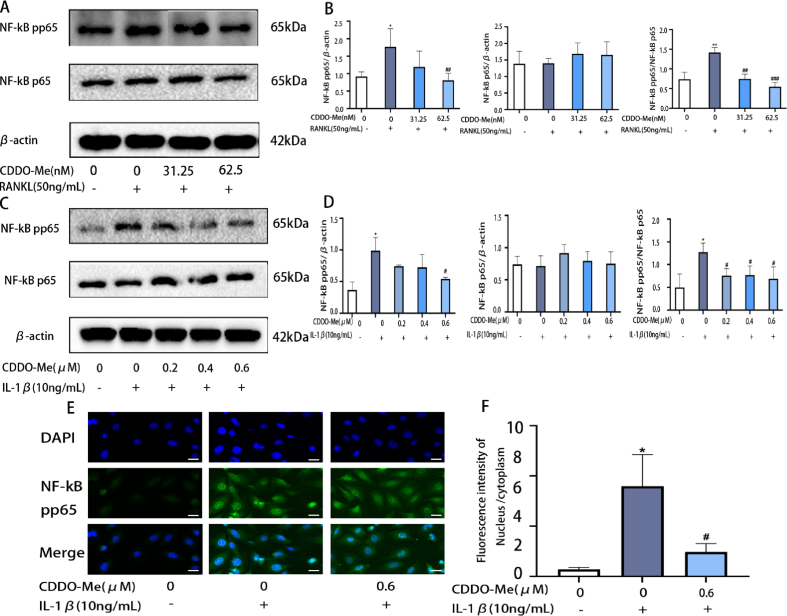
CDDO-Me inhibits with the NF-κB pathways in BMMs and ATDC5 cells. (A) Western blots analysis of NF-κB p65, p- NF-κB p65 in OCs pretreated with CDDO-Me (31.25 nM and 62.5 nM), followed by stimulation with or without RANKL (50 ng/mL) for 30min. (B) The quantification of NF-κB p65, p- NF-κB p65, theβ-actin was selected as the control protein, n = 3. (C) Western blots analysis of NF-κB p65, p- NF-κB p65 in ATDC5 pretreated with CDDO-Me (0.2 μM, 0.4 μM, and 0.6 μM), followed by stimulation with or without IL-1β (10 ng/mL) for 30 min. See supplementary material for non-adjusted images. (D) The quantification of NF-κB p65, p–NF–κB p65. The β-actin was selected as the control protein, n = 3. (E) Immunofluorescence staining was performed to analyze the nuclear translocation of p–NF–κB p65 upon IL-1βstimulation in ATDC5 cells, scale bar, 20 μm. (F) Nuclear fluorescence intensity/cytoplasmic fluorescence intensity. n = 3. *P < 0.05 and **P < 0.01 compared with control group. ^#^P < 0.05, ^##^P < 0.01 and ^###^P < 0.001 compared with IL-1βgroup or RANKL group.

Activation of the NF-κB pathway is important in RANKL-induced osteoclastogenesis [38]. To investigate whether CDDO-Me can inhibit osteoclast formation by inhibiting the NF-κB pathway. As shown in the diagram, Western blot results suggested that phosphorylated P65 was raised by RANKL induction. Surprisingly, 31.25 nM and 62.5 nM CDDO-Me suppressed the expression levels of phosphorylated P65 compared with those in the RANKL-induced group (Fig. 4A and B) (pp65: RANKL groups, p = 0.0493; 62.5 nM, p = 0.0390; pp65/p65: RANKL groups, p = 0.0058; 31.25 nM, p = 0.0027; 62.5 nM, p = 0.0009).

Besides, the NF-κB signaling pathway, which is directly involved in the regulation of MMP expression, is essential for the regulation of inflammation [39]. Phosphorylated NF-κB enters the nucleus to exert its biological effects. To evaluate whether CDDO-Me can inhibit the NF-κB signaling pathway and thus alleviate the development of inflammation after IL-1β stimulation, we pretreated the cells with CDDO-Me and then induced them with IL-1β for 30 min. The Western blot results showed that phosphorylated P65 was upregulated by IL-1β induction. In contrast, the phosphorylation of P65 was inhibited by CDDO-Me pretreatment (Fig. 4C and D) (pp65: IL-1β groups, p = 0.0112; 0.6 μM, p = 0.0203; pp65/p65: IL-1β groups, p = 0.0196; 0.2 μM, p = 0.0239; 0.4 μM, p = 0.0371; 0.6 μM, p = 0.0362). In addition, the immunofluorescence results showed that the expression of phosphorylated P65 in the nucleus was higher after IL-1β-stimulation. However, pretreatment with CDDO-Me can inhibit IL-1β-induced entry of phosphorylated P65 into the nucleus (Fig. 4E and F) (IL-1β groups, p = 0.0101; 0.6 μM, p = 0.0246).

These findings suggested that CDDO-Me suppressed the NF-κB pathway and inhibited RANKL-induced osteoclastogenesis and IL-1β-induced inflammatory response.

### 3.5 CDDO-Me suppresses RANKL-induced osteoclastogenesis and IL-1β-induced oxidative stress via the Nrf2/HO-1 pathway

**Fig. 5:**
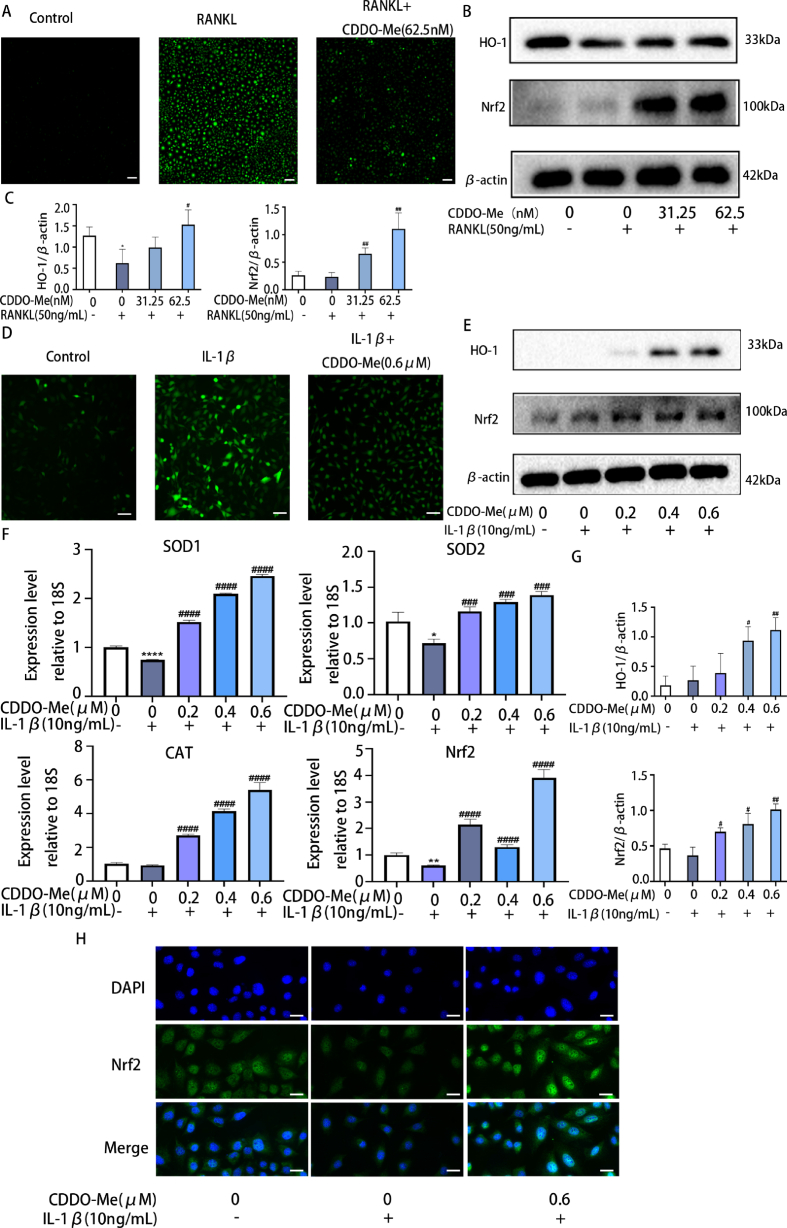
CDDO-Me suppresses RANKL-induced osteoclastogenesis and IL-1β-induced oxidative stress via the Nrf2/HO-1 pathway. (A) Detection of BMMs intracellular ROS production by DCFH-DA probe, scale bar, 100 μm. (B) Western blots analysis the expression of Nrf2 and HO-1 in OCs treated with CDDO-Me (31.25 nM and 62.5 nM) for 5 days and stimulation with or without RANKL (50 ng/mL) and the quantification of Nrf2 and HO-1(C). The β-actin was selected as the control protein, n = 3. (D) Detection of ATDC5 intracellular ROS production by DCFH-DA probe, scale bar, 50 μm. (E) Western blot analysis the expression of Nrf2 and HO-1 in ATDC5 pretreated with CDDO-Me (0.2 μM, 0.4 μM, and 0.6 μM) for 2 h, followed by stimulation with or without IL-1β (10 ng/mL) for 30min. See supplementary material for non-adjusted images. (F) oxidation-associated genes Sod1, Sod2, Cat and pathway gene Nrf2 were tested by qRT–PCR, n = 3. (G) The quantification of Nrf2 and HO-1. The β-actin was selected as the control protein, n = 3. (H) Immunofluorescence staining was performed to analyze the nuclear translocation of Nrf2 with IL-1β stimulation in ATDC5 cells, scale bar, 20 μm *P < 0.05 compared with control group. ^#^P < 0.05 and ^##^P < 0.01compared with RANKL group or IL-1βgroup.

ROS regulate osteoclast differentiation as intracellular signaling molecules [40]. To examine the suppressive effects of CDDO-Me on ROS levels, DCFH-DA staining was used to examine ROS levels in BMMs. The results showed that 50 ng/mL RANKL induced excessive ROS generation in BMMs. CDDO-Me 62.5 nM effectively reduced ROS levels (Fig. 5A). These results demonstrated that CDDO-Me effectively inhibited excessive ROS generation after RANKL treatment. The Nrf2/HO-1 pathway functions in osteoclast differentiation by regulating intracellular ROS [40]. We investigated whether CDDO-Me regulates RANKL-induced osteoclast formation through the Nrf2/HO-1 pathway. Fig. 5B Western blot shows that 31.25 nM and 62.5 nM CDDO-Me increased the expression levels of Nrf2 and HO-1 compared with those in the RANKL-induced group, and this result was consistent with the fold change data (Fig. 5C) (HO-1:0 nM, p = 0.0424; 62.5 nM, p = 0.0297; Nrf2: 31.25 nM, p = 0.0055; 62.5 nM, p = 0.0073).

In addition, excess production of reactive oxygen species (ROS) is closely related to OA [41]. ROS levels were detected by DCFH-DA staining. CDDO-Me (0.6 μM) effectively reduced ROS levels compared with 10 ng/mL IL-1β stimulated ATDC5 cells (Fig. 5D). The following oxidation-associated genes Sod1, Sod2 and Cat were tested by RT–qPCR in ATDC5. These genes were observably decreased by IL-1β. Conversely, the expression of these genes was significantly increased by CDDO-Me treatment compared with that in the IL-1β-induced group (Fig. 5F) (SOD1: all group, p < 0.0001; SOD2: IL-1β groups, p = 0.0208; 0.2 μM, p = 0.0009; 0.4 μM, p = 0.0001; 0.6 μM, p = 0.0001; CAT: all group, p < 0.0001; Nrf2: IL-1β groups, p = 0.0013; 0.2 μM, p < 0.0001; 0.4 μM, p < 0.0001; 0.6 μM, p < 0.0001). Nrf2/HO-1 pathway protein was detected, including Nrf2 and HO-1. Western blot analysis was used to evaluated the levels of Nrf2 and HO-1. The results showed that 0.2 μM, 0.4 μM and 0.6 μM CDDO-Me significantly elevated Nrf2 and HO-1 levels compared to those in the IL-1β group (Fig. 5E, G) (HO-1: 0.4 μM, p = 0.0257; 0.6 μM, p = 0.0094; Nrf2: IL-1β groups, p = 0.0424; 0.6 μM, p = 0.0297). This finding was similar to the RT-qPCR (Fig. 5F). In addition, immunofluorescence was used to examine the localization of Nrf2. The IL-1β-induced group exhibited reduced nuclear expression of Nrf2, while 0.6 μM CDDO-Me increased the expression of Nrf2 (Fig. 5H. These results suggested that CDDO-Me activated the Nrf2/HO-1 signaling pathway to suppressed OC differentiation and exerted antioxidant effects.

### 3.6 CDDO-Me attenuates the deterioration of human articular cartilage ex-vivo

**Fig. 6:**
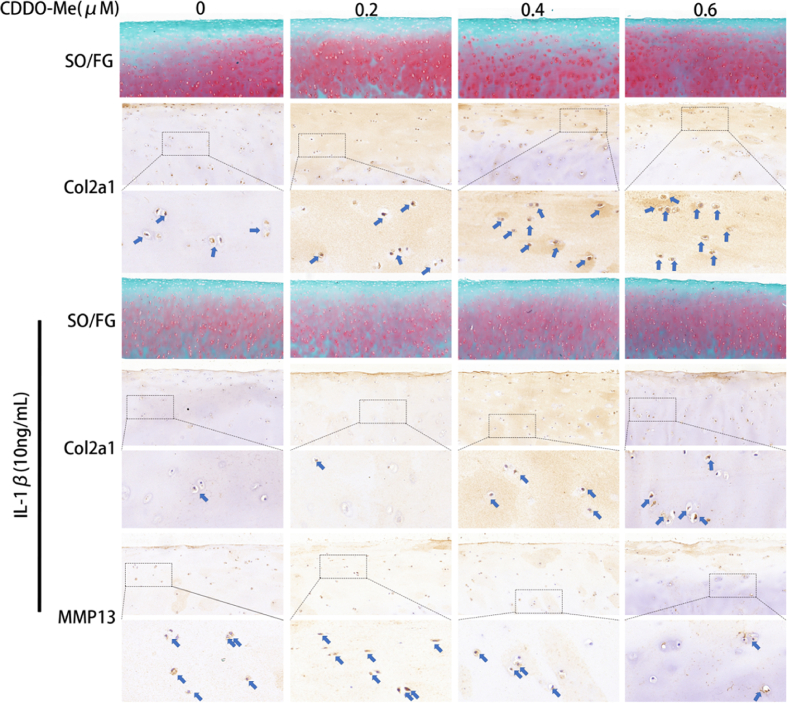
CDDO-Me attenuates the deterioration of human articular cartilage in vitro. Safranin O staining after treated with CDDO-Me (0.2 μM, 0.4 μM, and 0.6 μM) and with or without IL-1β, scale bar, 100 μm. Immunohistochemical staining of Col2a1 and MMP-13 with or without IL-1β, n = 3.

To further investigate the effects of CDDO-Me on human articular cartilage, we used an in vitro OA model of human articular cartilage induced by IL-1β for 72 h. Safranin O/Fast Green staining was performed; the lighter staining in the IL-1β-induced group than in the normal group indicated that IL-1β accelerated the degradation of the extracellular matrix. Conversely, 0.6 μM CDDO-Me induced a darker red color than 0.2 μM or 0.4 μM, indicating that 0.6 μM CDDO-Me was effective in inhibiting extracellular matrix degradation. Immunohistochemistry further revealed significantly increased expression of collagen II in CDDO-Me-treated cartilage explants compared to cartilage explants in the control or IL-1β groups. CDDO-Me also decreased expression of MMP13 (Fig. 6). These results indicated that CDDO-Me alleviated deterioration of the extracellular matrix in human articular cartilage.

### 3.7 CDDO-Me prevents subchondral bone loss and alleviates OA development in DMM mice

**Fig. 7:**
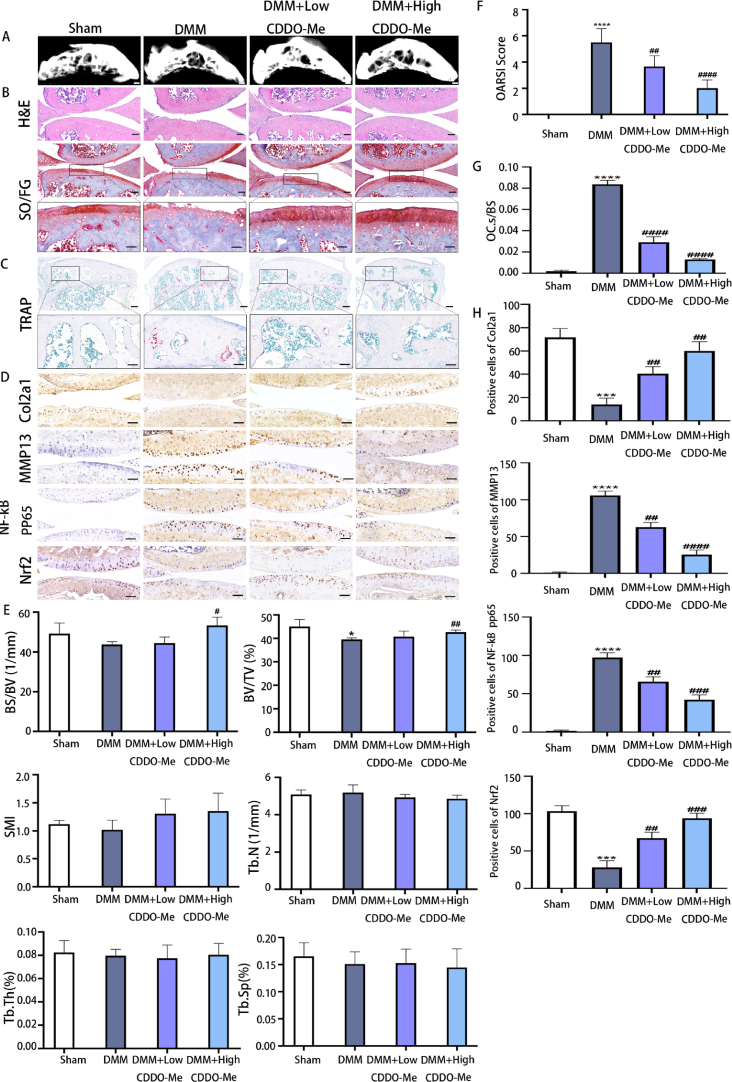
CDDO-Me prevents subchondral bone loss and alleviates OA development in DMM mice, n = 6. (A) 3D micro-CT images of subchondral bone after sham operation or DMM surgery, scale bar, 1 mm. (B) HE staining, scale bar, 100 μm. Safranin O staining, scale bar, 100 μm (top panelsnjjj); 50 μm (bottom panels). (C) Tartrate-resistant acid phosphatase (TRAP) staining, scale bar, 100 μm (top panels); 50 μm (bottom panels). (D) Immunohistochemical staining of Col2a1, MMP-13, p–NF–κB p65 and Nrf2 expression, scale bar, 50 μm. (E) Quantitative micro-CT analysis of tibial subchondral bone of bone volume fraction (BV/TV), bone surface/volume ratio (BS/BV), structure model index (SMI), trabecular number (Tb.N), subchondral trabecular thickness (Tb.Th), and trabecular separation (Tb.Sp). (F) Osteoarthritis Research Society International (OARSI) score indicates cartilage degeneration after the DMM operation. (G) Quantitative analysis of osteoclast surface per millimeter bone perimeter (Oc.s/BS) indicates subchondral bone remodeling. (H)The quantitative analysis of positive cells of Col2a1, MMP-13, p–NF–κB p65 and Nrf2 in each group. Low CDDO-Me (10 mg.kg-1), High CDDO-Me (50 mg.kg-1). *P < 0.05, ***P < 0.01 and ****P < 0.0001 compared with sham group. ^#^P < 0.05, ^##^P < 0.01, ^###^P < 0.001 and ^####^P < 0.0001 compared with DMM group.

To evaluate the effect of CDDO-Me in vivo, we conducted animal experiments. Mice with DMM-induced osteoarthritis were treated with different concentrations of CDDO-Me, and the tibias were isolated. To detect changes in subchondral bone in DMM mice treated with CDDO-Me, micro–computed tomography (micro-CT) analysis of the subchondral region was performed, and the results demonstrated significant collapse of the subchondral bone in the OA group (Fig. 7A). Compared to mice in the OA group, DMM mice treated with 50 mg kg^−1^ CDDO-Me showed less subchondral bone collapse and higher subchondral bone volume density (BV/TV) and bone surface/volume ratio (BS/BV). These OA-associated subchondral abnormalities were attenuated by CDDO-Me treatment. These results indicated that CDDO-Me might improve OA and prevent bone resorption. On the other hand, CDDO-Me treatment had no effect on the structure model index (SMI), trabecular number (Tb.N), subchondral trabecular thickness (Tb.Th), or trabecular separation (Tb.Sp) (Fig. 7E) (BV/TV: DMM groups, p = 0.0121; DMM + High CDDO-Me, p = 0.0010; BS/BV: DMM + High CDDO-Me, p = 0.0185).

The histo-morphological analysis results further supported the previous discoveries. As shown in Figure B, H&E and Safranin O-Fast Green staining showed smooth and dark red cartilage surfaces in the sham group, while the OA group exhibited fewer chondrocytes, cartilage damage, and massive proteoglycan degradation. Treatment with 50 mg kg^−1^ CDDO-Me improved these changes and slowed the progression of OA in DMM mice. Consistent with these data, the OARSI scores were smaller than those of the OA group (Fig. 7F) (DMM groups, p < 0.0001; DMM + Low CDDO-Me, p = 0.0070; DMM + High CDDO-Me, p < 0.0001).

The size of the red area (TRAP staining) represents the number of osteoclasts. The results are shown in Figure C, and the red area of the sham group was decreased, the area in the OA group was increased, and the red area was improved by 50 mg kg^−1^ CDDO-Me treatment compared with that in the OA group. The OC. S/BS results also indicated that CDDO-Me inhibited osteoclastogenesis in bone tissue (Fig. 7G). In addition, immunohistochemical analysis showed that MMP13-positive and PP65-positive cells were decreased, and Nrf2-positive and Col2-positive cells were increased by CDDO-Me treatment (Fig. 7D, H) (Col2a: DMM groups, p = 0.0004; DMM + Low CDDO-Me, p = 0.0053; DMM + High CDDO-Me, p = 0.0012; MMP13: DMM groups, p < 0.0001; DMM + Low CDDO-Me, p = 0.0011; DMM + High CDDO-Me, p < 0.0001; pp65: DMM groups, p < 0.0001; DMM + Low CDDO-Me, p = 0.0035; DMM + High CDDO-Me, p = 0.0004; Nrf2: DMM groups, p = 0.0004; DMM + Low CDDO-Me, p = 0.0048; DMM + High CDDO-Me, p = 0.0006). Overall, these results demonstrated that CDDO-Me prevented subchondral bone loss and protected against ECM degradation to alleviate OA development related to the NF-KB and Nrf2/HO-1 pathways in vivo.

## 4 Discussion

Osteoarthritis can affect the entire joint structure, such as articular cartilage, subchondral bone, and the synovium [42]. Local inflammation and oxidative damage are important factors that contribute to OA. Interventions targeting risk factors for OA offer a potential strategy to prevent OA progression. CDDO-Me was confirmed to exert anti-inflammatory and antioxidant effects [33]. We have shown that CDDO-Me prevents articular cartilage degradation by inhibiting extracellular matrix degradation and maintains normal subchondral bone structure by inhibiting osteoclast activity.

Nrf2 is a major regulator of cellular redox homeostasis. Nrf2 plays a defender role in the progression of OA, Nrf2 enters the nucleus to bind to AREs and activate the expression of downstream antioxidant genes, including HO-1, SODs, CAT, reduce ROS levels and inhibits the entry of p65 into the nucleus to reduce inflammation eventually suppression IL-1β-induced MMP-3 and MMP-13 expression [20,23,24,43]. In our results, CDDO-Me treatment increased the expression and translocation of Nrf2 and the activity of HO-1, SOD2, CAT. Finally, CDDO-Me activated the Nrf2-mediated intracellular antioxidant system, which was consistent with studies of the antioxidant mechanism of CDDO-Me in chondrocytes [44].

ROS play an important role in both chondrocytes and osteoclasts. Low levels of ROS are important for normal osteoclastogenesis and osteoblastogenesis to maintain bone integrity; however, excessive ROS cause mitochondrial dysfunction and gene expression changes, resulting in cartilage degradation and abnormal changes in subchondral bone [24]. RANKL has been shown to recruit TRAF6 through the receptor RANK, while ROS induce the expression of TRAF6 and increase the expression of OC transcription factors, including NFATc1 and c-Fos, indicating that inhibiting ROS production inhibits osteoclastogenesis [45,46]. Wang made similar findings that osteogenesis was significantly reduced by inhibiting the production of ROS [47]. Apart from this, ROS are associated with oxidative stress and inflammatory responses in OA, and IL-1β-induced chondrocytes produce increased levels of ROS [48]. In our study, treatment with CDDO-Me decreased the expression of TNFRSF11, which encodes RANK, NFATc1 and c-Fos, and the level of ROS were decreased in osteoclast. Similar to this, pretreatment with CDDO-Me attenuated IL-1β-induced ROS production in chondrocytes. Similar to our results, some study found that osteoarthritis progression was significantly attenuated by pharmacological inhibition of ROS production [49,50].

NF-κB pathway is considered a crucial downstream factor in the RANKL-induced differentiation of OC and is critical for NFATc1 activation [46,51]. RANKL can induce the phosphorylation of IκB, and phosphorylated p65 enters the nucleus to activate NFATc1 expression [52]. In the meantime, NF-κB mediates chondrocyte catabolism in response to IL-1β stimulation, which contributes to the increased expression of MMP13 and ADAMTS-5, resulting in ECM damage [53]. During the IL-1β-induced inflammatory response, phosphorylated p65 enters the nucleus to activate the expression of proinflammatory factors, thereby promoting OA [54]. Our study showed that CDDO-Me inhibited the phosphorylation and nuclear translocation of p65, and osteoclast fusion and resorption genes, including CTSK, Integrinβ3 and DC-STAMP are under the transcriptional control of NFATc1 and were subsequently reduced by CDDO-Me, eventually, osteoclast formation was significantly inhibited. Similar results were obtained in a study by Zhu in which osteoclast formation was inhibited by inhibiting the NF-κB pathway [52]. Besides, CDDO-Me decreased the expression of MMP3, MMP13, ADAMTS5, and increased the expression of aggrecan and Sox9, in response to the IL-1β-induced inflammatory response. But, the differential expression of MMP3 on protein and mRNA after CDDO-Me treatment may be due to increased degradation of MMP3 after translation into protein, suggesting to us that CDDO-Me may promote degradation of ECM catabolic protein and thus inhibit cartilage destruction during OA.

These results indicated that CDDO-Me inhibited NF-κB pathway by activating the Nrf2/HO-1 pathway to suppress the production of ROS, thereby inhibiting osteoclastogenesis and protecting chondrocytes from oxidative damage.

To further explore the protective effects of CDDO-Me in vivo, the DMM model was used to simulate the progression of osteoarthritis. Chondrocyte reductions, matrix degradation and cartilage ulceration were observed in the OA group by H&E, Safranin O-Fast Green and immunohistochemical (MMP13, Col2a1) staining. Intraperitoneal injection of 50 mg kg^−1^ CDDO-Me ameliorated these changes, and the effects on IL-1β-induced human cartilage explants were consistent with this result. Furthermore, the micro-CT results showed that the BV/TV was significantly increased by CDDO-ME treatment, and the number of TRAP-positive osteoclasts was significantly reduced, indicating that CDDO-ME could inhibit the differentiation of osteoclasts and reduce bone loss. The immunohistochemical results of PP65 and Nrf2 were consistent with the Western blot results and showed that Nrf2 expression increased, while p65 phosphorylation was inhibited.

Moreover, there are several limitations to our study. The effects of CDDO-Me should be tested in Nrf2-knockout mice. Our study has shown that CDDO-Me inhibited inflammation and oxidative stress in chondrocytes and inhibition osteoclastogenesis, but, more in-depth validation of relevant pathways is needed. Therefore, this is yet to be determined in future studies.

In summary, we showed that CDDO-Me treatment effectively inhibited ROS production and NF-κB pathway activation by activating the Nrf2/HO-1 signaling pathway and inhibited OA caused by enhanced osteoclast activity and accelerated degradation of chondrocyte extracellular matrix.

## 5 Conclusion

Taken together, there results suggest that CDDO-Me is a potential drugs for attenuating OA.

## Ethical review statement

This study was approved by Shanxi Medical University.

## Author contribution statement

Ruijia Yang: Conceived and designed the experiments; Performed the experiments; Contributed reagents, materials, analysis tools or data; Wrote the paper.

Yanjing Guo: Conceived and designed the experiments; Performed the experiments; Contributed reagents, materials, analysis tools or data.

Sujing Zong, Zhou Ma, Zhenyu Wang, Jiyu Zhao: Performed the experiments.

Jinmei Yang, Liping Li: Analyzed and interpreted the data.

Chongwei Chen: Conceived and designed the experiments.

Shaowei Wang: Conceived and designed the experiments; Contributed reagents, materials, analysis tools or data.

## Funding statement

Shaowei Wang was supported by 10.13039/501100001809National Natural Science Foundation of China [81572207 & 81871815], Health Commission of Shanxi Province [2020075 & 2020XM54].

## Data availability statement

Data included in article/supp. material/referenced in article.

## Declaration of interest's statement

The authors declare that they have no known competing financial interests or personal relationships that could have appeared to influence the work reported in this paper.
