# Supplementary material for: Bardoxolone methyl ameliorates osteoarthritis by inhibiting osteoclastogenesis and protecting the extracellular matrix against degradation
Source: Heliyon. 2023 Jan 20;9(2):e13080. doi: 10.1016/j.heliyon.2023.e13080 (PMC9925876; doi:10.1016/j.heliyon.2023.e13080)
Supplement: Multimedia component 1 [file mmc1.docx]

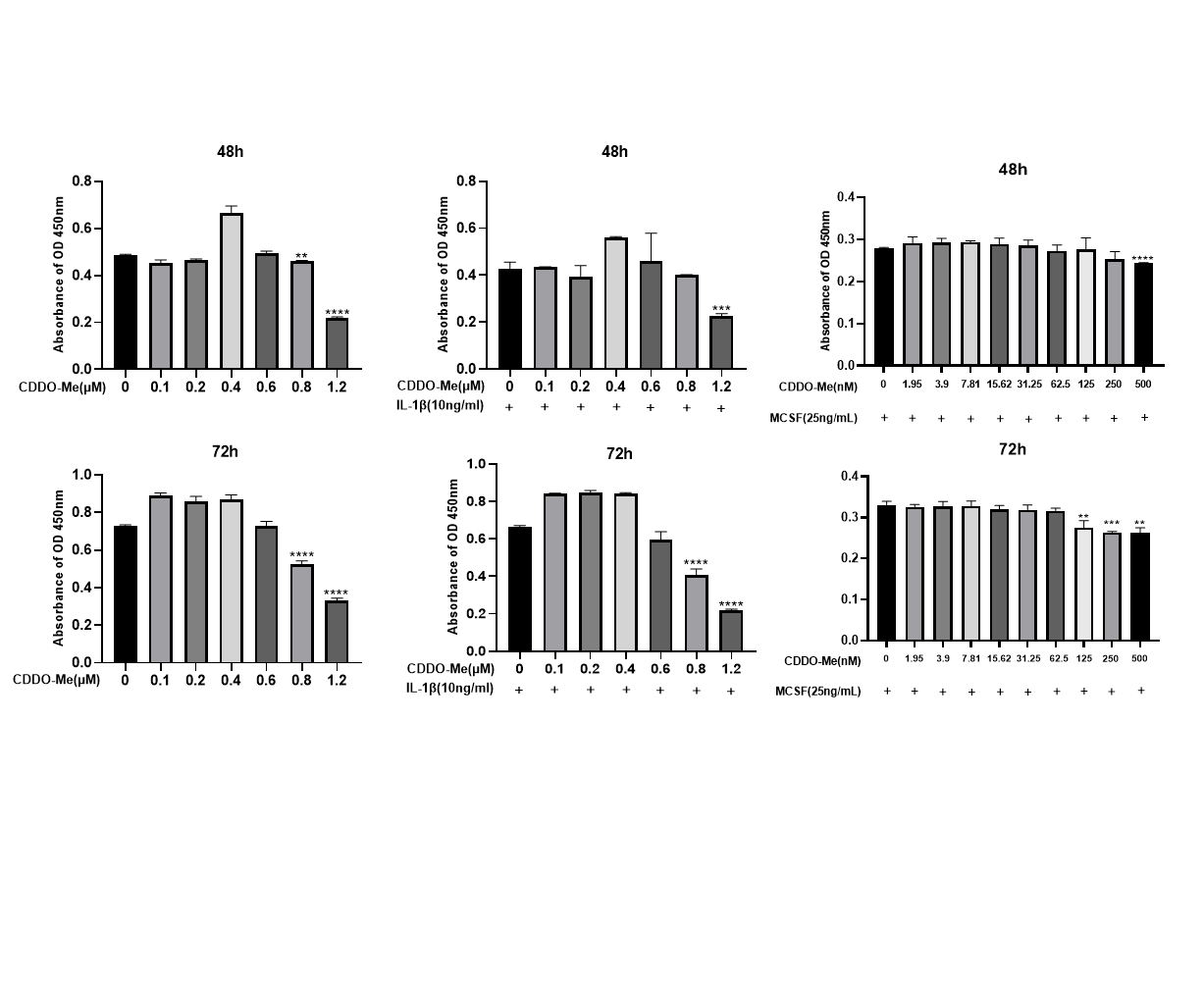


B

A

S1：（A）Assesses of the cytotoxicity of CDDO-Me on ATDC5 by the CCK-8 assay at 48h and 72h.（B）Assesses of the cytotoxicity of CDDO-Me on BMMs by the CCK-8 assay at 48h and 72h.
