# Supplementary material for: Bardoxolone methyl ameliorates osteoarthritis by inhibiting osteoclastogenesis and protecting the extracellular matrix against degradation
Source: Heliyon. 2023 Jan 20;9(2):e13080. doi: 10.1016/j.heliyon.2023.e13080 (PMC9925876; doi:10.1016/j.heliyon.2023.e13080)

2B

c-Fos


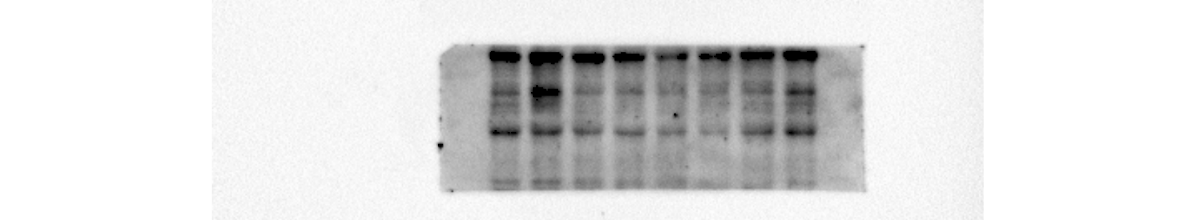


CTSK


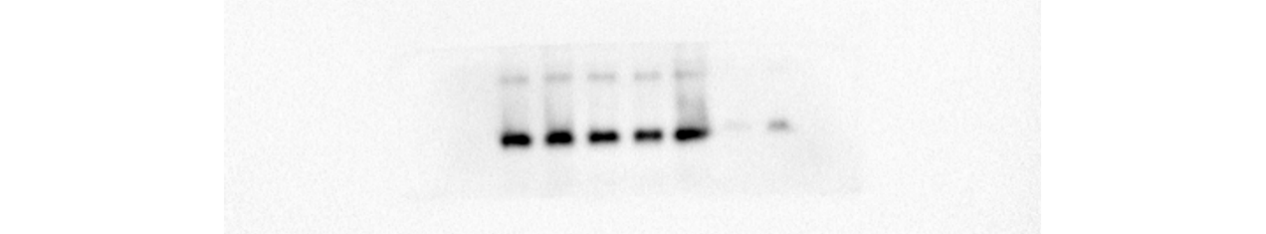


Integrin β3


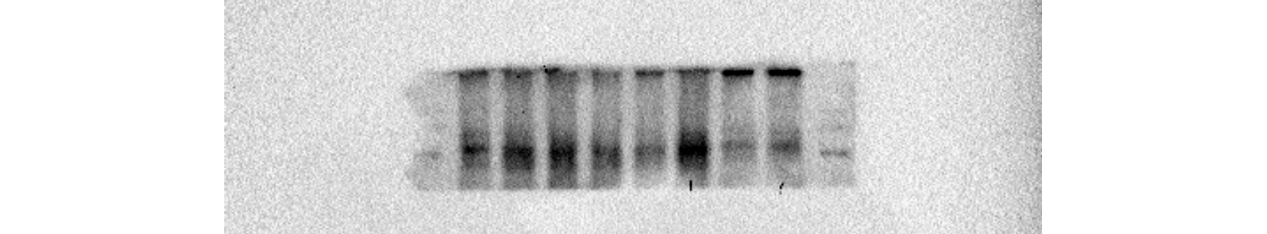


NFATc1


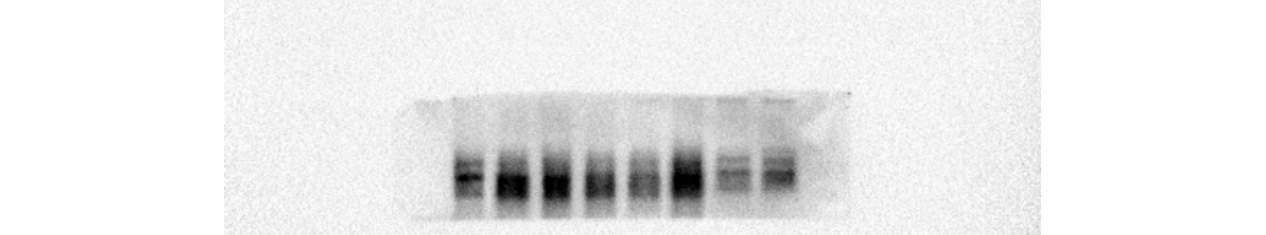


β-actin


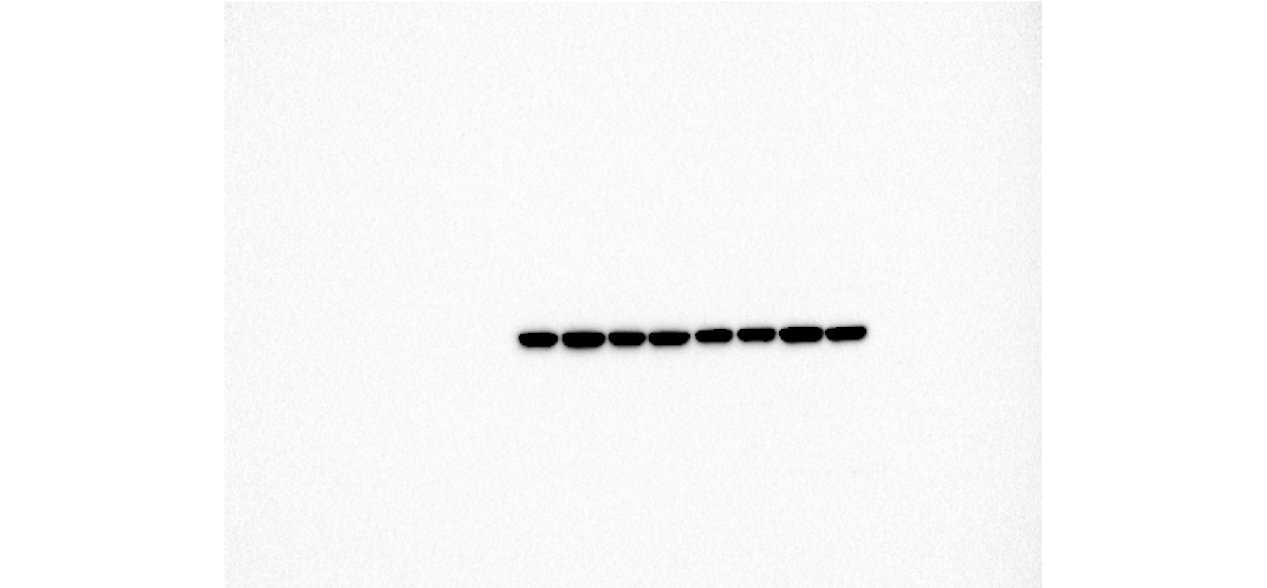


3E

MMP13


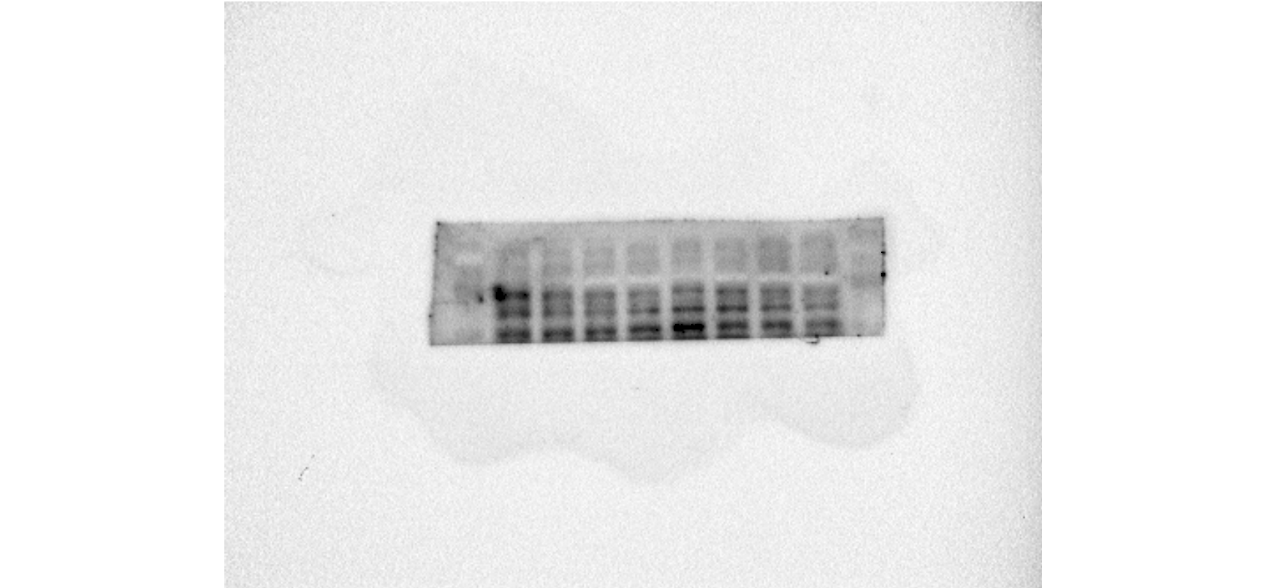


MMP3


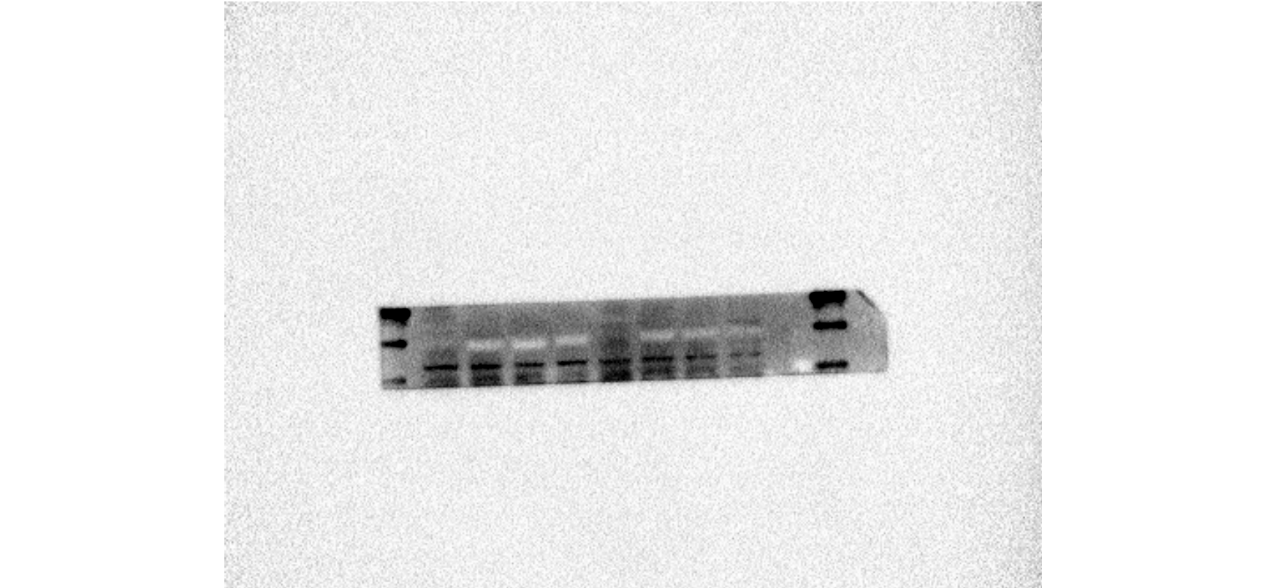


Aggrecan


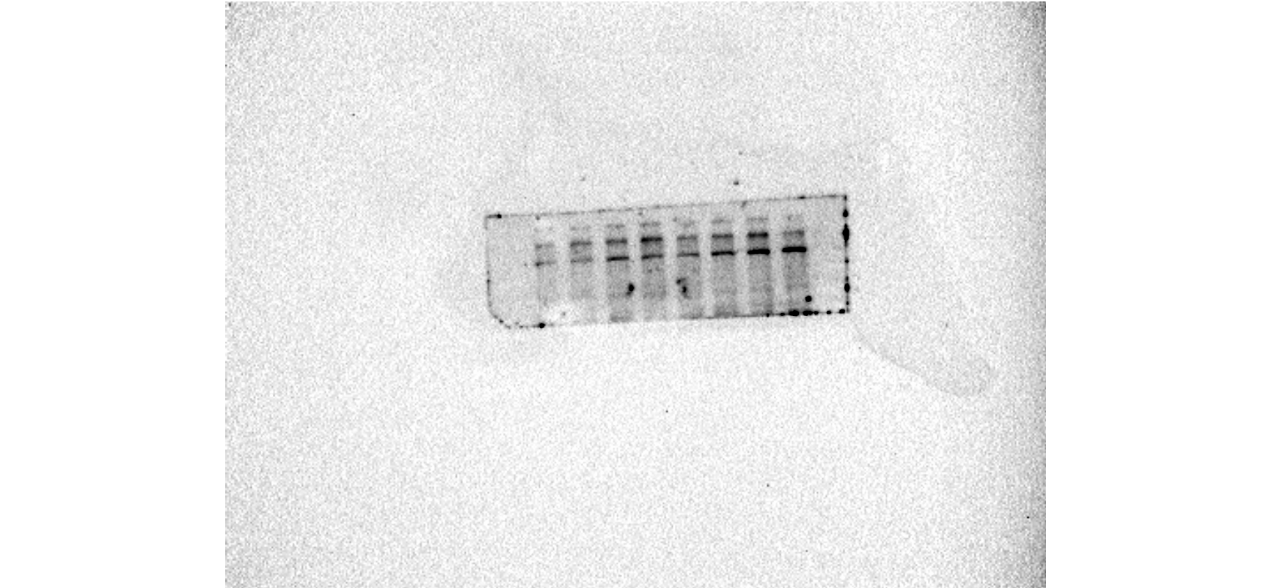


Sox9


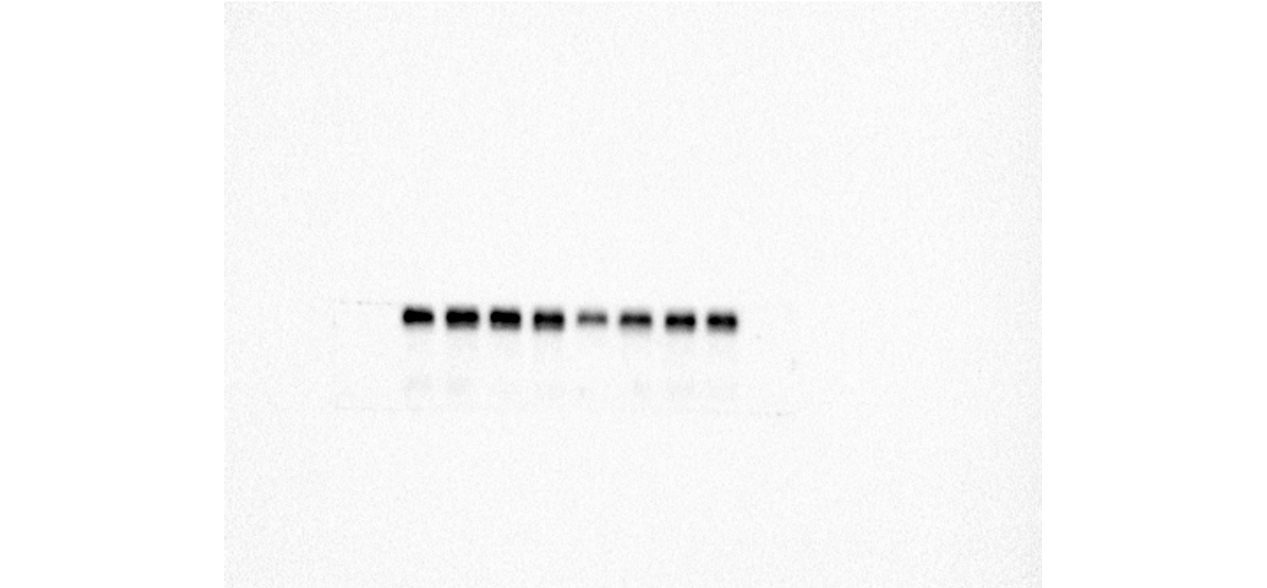


GAPDH


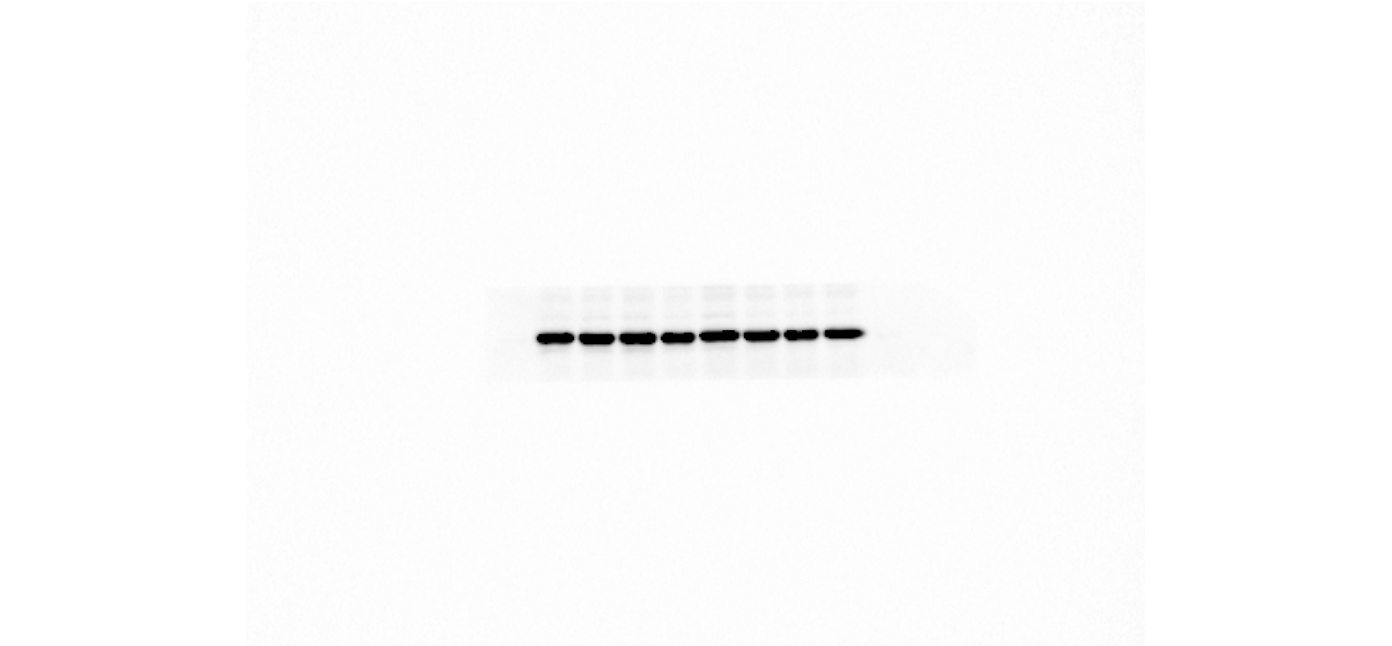


4A

NF-kB pp65


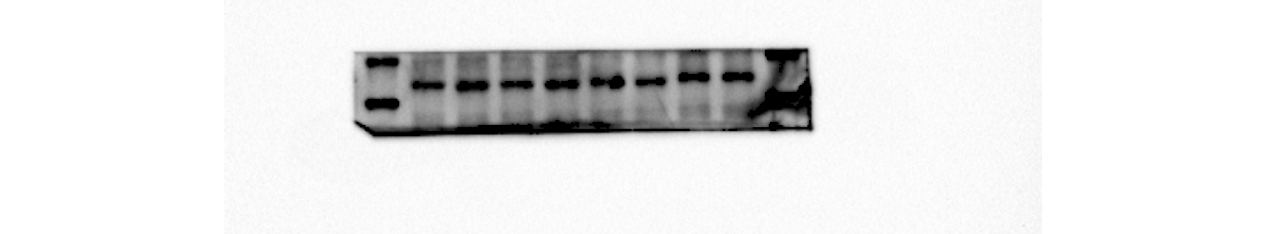


NF-kB p65


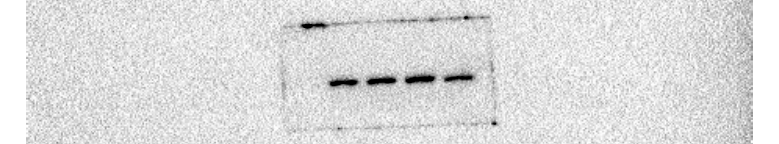


β-actin


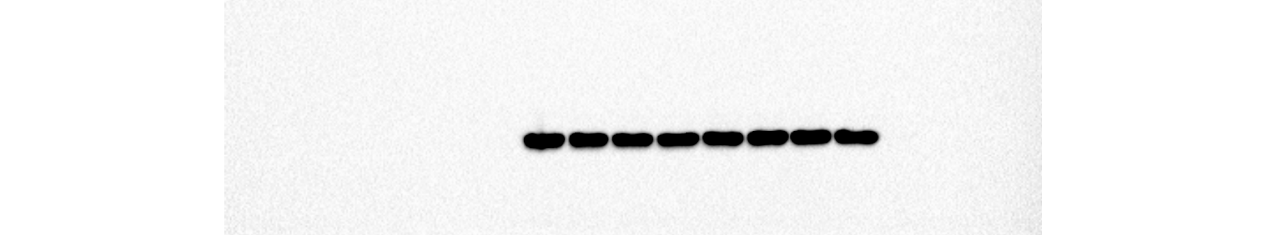


4C

NF-kB pp65


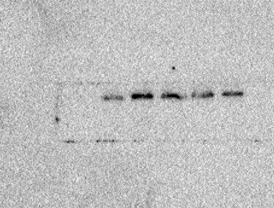


NF-kB p65


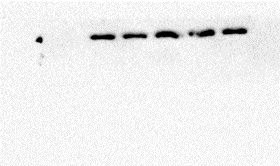


β-actin


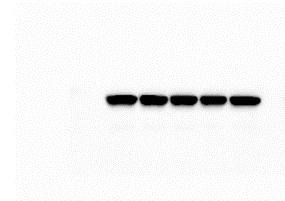


5B

HO-1


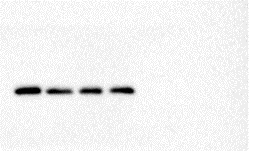


Nrf2


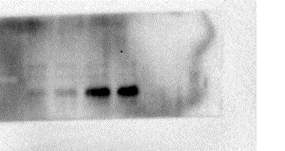


β-actin


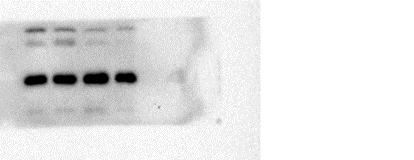


5E

HO-1


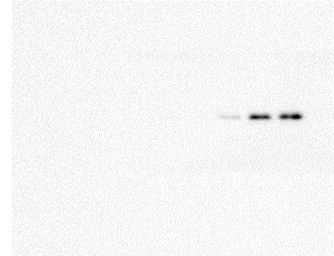


Nrf2


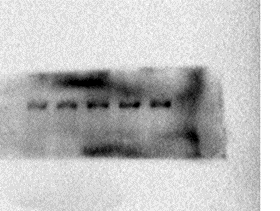


β-actin


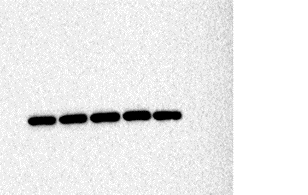

Supplement: Multimedia component 2 [file mmc2.docx]
